# A novel machine-learning-derived genetic score correlates with measurable residual disease and is highly predictive of outcome in acute myeloid leukemia with mutated *NPM1*

**DOI:** 10.1038/s41408-019-0244-2

**Published:** 2019-10-01

**Authors:** Nikhil Patkar, Anam Fatima Shaikh, Chinmayee Kakirde, Shrinidhi Nathany, Hridya Ramesh, Prasanna Bhanshe, Swapnali Joshi, Shruti Chaudhary, Sadhana Kannan, Syed Hasan Khizer, Gaurav Chatterjee, Prashant Tembhare, Dhanalaxmi Shetty, Anant Gokarn, Sachin Punatkar, Avinash Bonda, Lingaraj Nayak, Hasmukh Jain, Navin Khattry, Bhausaheb Bagal, Manju Sengar, Sumeet Gujral, Papagudi Subramanian

**Affiliations:** 1 Haematopathology Laboratory, ACTREC, Tata Memorial Centre, Navi Mumbai, Maharashtra India; 2 Biostatistics, ACTREC, Tata Memorial Centre, Navi Mumbai, Maharashtra India; 3 Adult Haematolymphoid Disease Management Group, Tata Memorial Centre, Mumbai, Maharashtra India; 4 Department of Cytogenetics, ACTREC, Tata Memorial Centre, Navi Mumbai, Maharashtra India

**Keywords:** Cancer genomics, Translational research

Dear Editor,

Acute myeloid leukemia with mutated *NPM1* (*NPM1*^mut^ AML), one of the commonest subtypes of AML, is characterized by a favorable outcome in the absence of accompanying *FLT3*-internal tandem duplications (ITD)^1^. *NPM1*^mut^ AML has a high degree of mutational heterogeneity and harbors an average of 3–4 mutations per case (most commonly involving genes implicated in DNA methylation, cell signaling, cohesin complex, and RNA splicing)^2^. Due to advances in sequencing technologies, we now recognize that age-related clonal hematopoiesis (ARCH) is a possible precursor to myeloid malignancies, such as myelodysplastic syndromes and AML. Mutations in genes such as *DNMT3A*, *TET2*, and *ASXL1* account for >90% of ARCH mutations in AML^3^. Interestingly, mutations in these ARCH-defining genes are also harbored by *NPM1*^mut^ AML indicating a putative synergistic mechanism in contributing to leukemogenesis^2^. In that context, variant allele fractions (VAF) generated through next-generation sequencing (NGS) data sets are informative in recreating clonal hierarchy of a tumor sample. By using this information, we can distinguish founder mutations (which would have a higher VAF) from sub-clonal mutations that arise subsequently^4^.

Although NGS technologies have produced a deluge of cancer genomics data, it is challenging to accurately predict disease outcome from these data sets. Machine learning (ML), a branch of artificial intelligence, has shown tremendous potential toward interpretation of complex genomic data sets^5^. By using ML, researchers are now able to discover novel patterns between data and use this information for predicting cancer susceptibility, recurrence, prognostication, and therapy^6^. In addition, ML has also been used to predict transplant-related mortality with considerable success^7^. In a proof of concept, we used a supervised ML approach to identify clinically important genomic aberrations in *NPM1*^mut^ AML. Based on these data, we developed a scoring model that provides a mechanism to risk stratify *NPM1*^mut^ AML, a seemingly homogeneous disease entity.

A total of 110 patients (Supplementary Table 1) of adult (≥18 years) *NPM1*^mut^ AML were accrued over a 6-year period from March 2012 to December 2018. The median follow-up for our cohort was 26.8 months. The mean OS was 46.7 months (median not reached; 95% CI: 40–53.5) and mean RFS was 44.9 months (95% CI: 37.8–52.0). These patients were sequenced by using a 50-gene panel composed of 1066 single-molecule molecular inversion probes (smMIPS) on an Illumina MiSeq sequencer^8^. Additional details pertaining to design of the panel and data analysis are described in Supplementary Methods (Supplementary Table 2). A total of 389 somatic mutations (including those occurring in *NPM1* gene) were harbored by this cohort (Fig. 1).

**Fig. 1:**
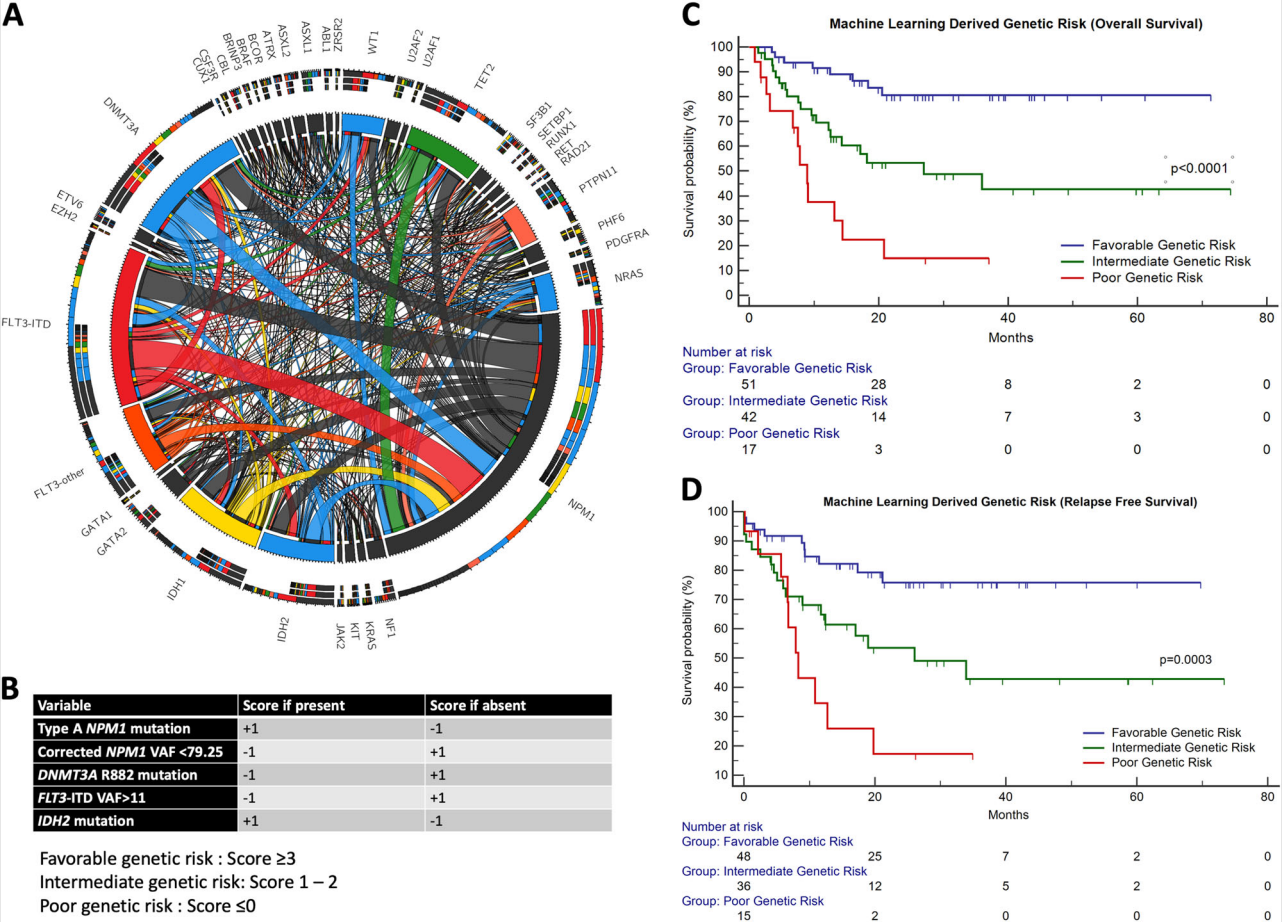
A total of 389 somatic mutations were harbored among 110 patients. The above circos plot (1 A) highlights the genomic complexity of *NPM1-*mutated AML. Commonly occurring gene mutations are colored. The scoring system is detailed in panel **b**. Clinical relevance of the machine-learning-derived genetic score for *NPM1-*mutated AML is also depicted here. The Kaplan–Meier plot in the top-right section (**c**) shows the clinical relevance of genetic risk when factored for overall survival (OS) and plot on the lower right (**d**) for relapse-free survival (RFS)

It is still unclear if *NPM1*^mut^ AML in which the *NPM1* gene per se is not a founder mutation has any different prognosis from the rest. To address this lacuna in the literature, we devised a new metric called corrected *NPM1* VAF where we compute the *NPM1* allelic abundance as a fraction of the largest VAF for that sample. For example, if *NPM1* was the highest VAF for a given sample the corrected VAF was 100%. Similarly, if *NPM1* VAF was 40% and another variant was at 50%, the corrected *NPM1* VAF was 80%. Based on receiver-operating characteristic (ROC) analyses, we determined that a corrected *NPM1* VAF cutoff value of ≤79.25 provided the optimal classification of patients as *NPM1* VAF high or low. A similar ROC analysis was done for *FLT3*-ITD VAF levels where VAF levels were computed against OS to classify patients as *FLT3*-ITD VAF high or low. For *FLT3*-ITD VAF, a cutoff of 11 helped classify patients as high *FLT3*-ITD VAF (>11) and the rest as low *FLT3*-ITD VAF.

The performance characteristics of the ML model are depicted in supplementary data (Supplementary Tables 3 and 4, Supplementary Fig. 1). Based on these data, the top five variables most likely to predict a patient to be alive were high corrected *NPM1* VAF, low *FLT3*-ITD VAF, presence of *IDH2* mutation, absence of *DNMT3A* R882 mutation, and type A *NPM1* mutation. A final score for that case was devised as a sum total of the individual scores. This score is elaborated in Fig. 1. Measurable residual disease (MRD) was assessed by using multiparametric FCM (FCM–MRD). Out of 100 patients who were in morphological remission, post-induction FCM–MRD assessment was performed in 99. Of these FCM–MRD was detected in 27.1%. The presence of FCM–MRD was predictive of an inferior OS (*p* = 0.007) and RFS (*p* = 0.01) as seen in Supplementary Data (Supplementary Fig. 2, Supplementary Table 5). A strong statistical correlation was observed between ML-derived genetic risk and post-induction FCM–MRD ((*p* = 0.001), Supplementary Fig. 3).

Patients who were classified as poor genetic risk had an inferior OS and RFS as compared with patients in favorable and intermediate risk classes (Fig. 1c, d; Supplementary Table 5). The results of univariate and multivariate Cox analysis are seen in Table 1. FCM–MRD as well as genetic risk were important determinants of outcome. On multivariate Cox analysis (Table 1), the presence of poor genetic risk was the most important independent factor when factored for OS as well as RFS.

**Table 1:** Prognostic significance of MRD, machine-learning-derived genetic risk in *NPM1-*mutated AML by univariate and multivariate Cox analysis

Univariate Cox analysis					
	Overall survival (OS)			Relapse-free survival (RFS)	
	HR (95% CI)		*p*	HR (95% CI)	*p*
Machine-learning-derived genetic risk					
Favorable genetic risk	1		<0.0001	1	0.0003
Intermediate genetic risk	3.54 (1.81–6.94)			2.71 (1.36–5.38)	
Poor genetic risk	8.57 (2.84–25.86)			5.19 (1.76–15.27)	
Post-induction FCM–MRD					
MRD negative	1		0.007	1	0.02
MRD positive	1.44 (0.9–2.3)			1.48 (1.0–2.18)	

Factors	HR	95% CI	*p*
*Multivariate Cox analysis: overall survival*			
Intermediate genetic risk	3.52	(1.42–8.78)	0.0071
Poor genetic risk	7.94	(3.0–21.35)	<0.0001
Post-induction FCM–MRD positive	1.62	(1.1–2.37)	0.015
*Multivariate Cox analysis: relapse-free survival*			
Intermediate genetic risk	2.38	(1.07–5.31)	0.03
Poor genetic risk	4.86	(1.98–11.97)	0.0006
Post-induction FCM–MRD positive	2.05	(1.01–4.15)	0.05

*MRD* measurable residual disease, *OS* overall survival, *RFS* relapse-free survival, *HR* hazards ratio, *CI* confidence interval

Genetic scoring systems have been used systematically for precursor B lineage acute lymphoblastic leukemia by incorporating copy-number alteration and cytogenetics data with great success^9^. Rather than focusing on individual risk factors, we predicted that a combinatorial approach was most likely to yield relevant prognostic information. This is evident by good correlation of genetic risk classes with FCM–MRD as well as clinical outcome. This study, to the best of our knowledge, represents a novel application of ML to *NPM1*^mut^ AML. Our data indicate that this scoring system will be useful in identifying *NPM1*^mut^ AML patients who are at high risk of relapse and distinguishes them from patients who are at truly good risk. In our data set, poor genetic risk patients had a much shorter survival as compared with patients in favorable genetic risk category (Fig. 1; Supplementary Table 5). Such patients will require intensive post-remission strategies, such as hematopoietic stem cell transplantation or experimental therapies.

Recently, Cappelli et al. in a large study on *NPM1*^mut^ AML demonstrated that *DNMT3A* R882 mutation was commonly seen in younger adults as compared with older patients^10^. Although our cohort is a young AML cohort, we found that these R882 mutations were almost equally distributed as compared with other *DNMT3A* mutations (15.5% as compared with 16.4%). In addition, we found that *DNMT3A* R882 mutations are associated with inferior outcome as opposed to other *DNMT3A* mutations (Supplementary Fig. 4).

Dunlap et al. recently demonstrated that *IDH* mutations in combination with *DNMT3A* mutations predict for an inferior outcome^11^. However, the clinical relevance of *IDH* mutations in AML is unclear due to conflicting data^4^^,11–13^. Our data indicate that *IDH2* (in our data set limited to *IDH2* R140 hotspot mutation) and *NPM1* co-mutated AML is a favorable disease entity especially in the context of other variables in the genetic scoring system proposed by us (Supplementary Fig. 5).

High allelic fractions of recurrently mutated genes in AML such as *FLT3* (namely *FLT3*-ITD) are associated with poor outcome^14^. Patel et al. described that high *NPM1* VAF levels had an association with poor outcome^15^. These findings were however refuted by another group^16^. Rather than analyzing upfront VAF levels, we devised a new metric called corrected *NPM1* VAF. Expectedly, cases where *NPM1* is not the early clone are dominated by ARCH mutations (Supplementary Fig. 6), and this may be an additional factor contributing to poor outcome. In fact, patients with low corrected *NPM1* VAF harbored higher frequencies of *IDH1* mutations as compared with the rest (Supplementary Fig. 7). On factoring in the type of *NPM1* mutation (type A or otherwise) based on existing literature, we determined that this was clinically relevant especially in the context of other ML-derived variables^17,18^.

To summarize, a supervised ML approach identified clinically important genomic aberrations in *NPM1*^mut^ AML. By using these data, we devise a scoring system that enables us to subclassify *NPM1*-mutated AML into three prognostic classes. We demonstrate a good correlation of this machine-learning-derived genetic score with FCM–MRD. Finally, we also show that ML-derived genetic risk classes have vastly differing outcomes, and these classes are independent predictors of clinical outcome. The limitations of our study include a relatively small cohort and retrospective analysis. The cutoffs for corrected *NPM1* and *FLT3*-ITD VAFs in this study will only be approximate in nature, and given the variability of different NGS methodologies as well as sequencing platforms, are likely to change. The scoring system as well as these cutoffs should be validated prospectively by other groups.

## Supplementary information

Supplementary Data

Supplementary Cytogenetics Data
