# Supplementary material for: A novel machine-learning-derived genetic score correlates with measurable residual disease and is highly predictive of outcome in acute myeloid leukemia with mutated NPM1
Source: Blood Cancer J. 2019 Oct 1;9(10):79. doi: 10.1038/s41408-019-0244-2 (PMC6773777; doi:10.1038/s41408-019-0244-2)
Supplement: Supplementary file 1 — Supplementary Data [file 41408_2019_244_MOESM1_ESM.pdf]

SUPPLEMENTARY METHODS

Patient Details:

A summary of clinical and laboratory parameters is depicted in supplementary Table 1 below.

a. Patient Accrual and Initial work up: The study was approved by the institutional ethics committee (TMC – IEC III Project 163). A total of 110 patients of adult (≥18 years) AML with mutated *NPM1* were accrued over a 6-year period from March 2012 to December 2018 after informed consent. The patients were diagnosed as per the standard WHO 2008 recommendations. Fluorescent in-situ hybridization (FISH) and karyotyping were done using standard techniques.(1)

b. Treatment of *NPM1*^{mut} AML and MRD Sampling: All patients were treated using conventional induction "3+7" chemotherapy consisting of daunorubicin (60 mg/m2 D1-D3) and cytarabine (100 mg/m2/day D1-D7). Complete remission (CR) was defined as a morphologic leukemia free state (<5% blasts in bone marrow /absence of circulating blasts) at end of induction therapy. At this point the first MRD sample was obtained [called post induction (PI) MRD]. If patients were in morphologic CR, they were further treated with three courses of 12-18 gm/m2 high dose cytarabine (HiDAC) or underwent allogeneic bone marrow transplantation (aBMT), if it was deemed feasible. All patients who were not in morphological remission discontinued treatment and went on palliation / supportive care. Of these, a single patient continued his treatment as per protocol as per discretion of treating physician. Only 7 patients (6.4%) out of the 110 analyzed here received allogeneic bone marrow transplantation (BMT). Patients receiving allogeneic BMT did not have a different OS (p=0.8) or RFS (p=0.93) as compared to the rest (presumably due to small numbers) and therefore were not considered separately.

c. Evaluation of Treatment Outcome: Overall survival (OS) and relapse free survival (RFS) were calculated as previously described.(2, 3) There were only 2 patients above 60 years. No difference in outcome was observed based on age for either OS or RFS.

Parameter	Observation (%)
**Demographics:**	
Age	Range: 19-62 years; Median: 41 years
Sex	Male: Female: 1.1:1
**Clinical Characteristics:**	
Total Number of Patients Accrued	110
Cases Not in Morphological Remission	10
**Remission Characteristics:**	
Complete remission (CR)	16
CR with incomplete hematologic recovery (CRi)	83
**Bone Marrow Transplantation:**	
Patients who underwent BMT	07
**Laboratory Characteristics:**	
Blood Counts at Presentation	108
1. More than 50,000/mm^3	29
2. Less than 50,000/mm^3	80
Post Induction FCM-MRD (for patients in morphological CR):	99
1. MRD Positive:	28 positive (28.3%)
2. MRD Negative:	71 negative (71.7%)
**Individual Parameters of Genetic Risk Score:**	
1. Type A *NPM1* mutation	78 (70.9%)
2. Corrected *NPM1* VAF<79.25	14 (12.7%)
3. *DNMT3A* R882 mutation present	17 (15.5%)
4. *FLT3*-ITD VAF > 11	22 (20.0%)
5. *IDH2* mutation	26 (23.6%)
**Classification according to Genetic Risk:**	
Favorable Genetic Risk (Fav-ML-GR)	51 (46.4%)
Intermediate Genetic Risk (Int-ML-GR)	42 (38.2%)
Poor Genetic Risk (Poor-ML-GR)	17 (15.5%)

Supplementary Table 1: Summary of Clinical and Laboratory Parameters

B. Genetic Testing on Diagnostic Sample:

a. Cytogenetics: A complete list of cytogenetic aberrations can be seen in supplementary file accompanying this manuscript.

b. Fragment length assay-based detection of FLT3-internal tandem duplications (*FLT3*-ITD): FLT3-ITD were detected using a fragment length assay as described previously. (3) A total of 52 patients (42.3%) were FLT3-ITD positive. We did not observe any statistically relevant difference with respect to OS (p=0.3) or RFS (p=0.4) between the FLT3-ITD positive and negative *NPM1*^{mut} AML cohorts.

c. Detection of somatic mutations using single molecule molecular inversion probes (smMIPS): We created a targeted resequencing panel for myeloid malignancies

comprising of 1066 smMIPS. This pool of smMIPS captures the coding regions (plus 5bp intronic flank) of genes implicated in the pathogenesis of myeloid malignancies as seen in supplementary table 2. The library was pooled and subsequently rebalanced to ensure similar capture efficiencies of targeted regions. 600ng of genomic DNA was captured with smMIPS panel, treated with exonucleases, PCR amplified and size selected to generate sequencing ready libraries that incorporated dual sample specific indices. This library was sequenced using 300 cycle Illumina MiSeq v2 chemistry (Illumina, San Diego, CA USA). Additionally, in every case, *CEBPA* gene indels and *NPM1* gene mutations were detected as described previously. (3)

**Name**	**RefSeq ID**	**Region**	**Name**	**RefSeq ID**	**Region**
ABL1	NM_007313	Exon4-8	*KRAS*	NM_004985	Exon2-5
ASXL2	NM_018263	Exon11-12	*MPL*	NM_005373	Exon4-12
ASXL1	NM_015338	Exon12-13	*NF1*	NM_001042492	Exon2-58
ATRX	NM_000489	Exon 4-35	*NPM1*	NM_002520	Exon11
BCOR	NM_001123383	Exon4-15	*NRAS*	NM_002524	Exon2-5
BRAF	NM_004333	Exon15	*PDGFRA*	NM_006206	Exon2-23
CALR	NM_004343	Exon9	*PHF6*	NM_032458	Exon2-9
CBL	NM_005188	Exon4-9	*PTEN*	NM_000314	Exon5-9
CDKN2A	NM_000077	Exon1-2	*PTPN11*	NM_002834	Exon3,13-14
CSF3R	NM_156039	Exon14-17	*RAD21*	NM_006265	Exon2-14
CUX1	NM_181552	Exon15-23	*RUNX1*	NM_001754	Exon4-9
DNMT3A	NM_022552	All Exons	*RET*	NM_020975.6	All Exons
EZH2	NM_152998.2	Exon2-19	*SF3B1*	NM_012433	Exon6-25
ETV6	NM_001987	Exon1-8	*SH2B3*	NM_005475	Exon2-8
ETNK1	NM_018638	Exon3	*SMC1A*	NM_006306	Exon2-23
FAM5C	NM_199051	Exon3-7	*SRSF2*	NM_003016	Exon1-2
FLT3	NM_004119	Exon14-16,20	*SETBP1*	NM_015559	Exon2-6
GATA1	NM_002049	Exon2	*STAG2*	NM_006603	Exon5-30
GATA2	NM_032638	Exon2-6	*TET2*	NM_001127208	Exon3-11
IDH1	NM_005896	Exon4	*TP53*	NM_001126114	All Exons
IDH2	NM_002168	Exon4	*U2AF1*	NM_006758	Exon2-8
JAK2	NM_004972	Exon11-21	*U2AF2*	NM_007279	Exon5-6,11
JAK3	NM_000215	Exon11,13,15	*WT1*	NM_024426	Exon7,11
KDM6A	NM_021140	Exon2-29	*ZBTB7A*	NM_015898	Exon2-3
KIT	NM_000222	Exon2-3,7-18	*ZRSR2*	NM_005089	Exon2-11

Supplementary Table 2: List of genes and their loci sequenced using the targeted myeloid sequencing panel.

d. Data Analysis Pipeline and Filtering of Variants: Fastq files were generated using the MiSeq instrument demultiplexing software. Paired end assembly was performed using PEAR (v0.9.8). The assembled reads were mapped to the human genome (build hg19)

using bwa aligner (v0.7.17) and further preprocessed using Picard (v2.1.1) and samtools (v1.3.1). Alignment files were further processed using different GATK (v3.8) walkers (RealignerTargetCreator, IndelRealigner, BaseRecalibrator). A .mpileup file was generated using samtools (v1.3.1). Variant calling was performed using Mutect2, Platypus (v.0.8.1) and Varscan2 (v.2.3.9). *FLT3*-internal tandem duplications were additionally detected using ITDSeek. Variant files were annotated using annovar with population frequency databases (1000Genomes, Exome Aggregation Consortium datasets) as well as the Cosmic database (Cosmic v83). This data was processed and collated with internal scripts. Variants were filtered by focusing on exonic regions (including splicing variants if any) followed by population frequency (<0.01) filtering. This was followed by in-silico prediction using (SIFT, PolyPhen2, CADD, PROVEAN, MutationTaster, MutationAssessor, M-CAP, FATHMM, LRT, DANN). *FLT3*-ITD was called only if it could be confirmed using fragment length analysis. ITDSeek variants were called that had a quality score > 20. AMP/ACMG guidelines were used for reporting of variants. (4) For multiple *FLT3*-ITD a summation of the variant allele fraction (VAF) levels was done. The median coverage was 1081x (range: 131-3925)

e. <u>Machine Learning & Genetic Score:</u> For feature selection we selected gene mutations occurring at a frequency of ≥ 10% in *NPM1* mutated AML as well as the corrected *NPM1* and *FLT3*-ITD VAF classes (after ROC based classification). *DNMT3A* mutations were divided into R882 and non-R882 *DNMT3A* mutations. Similarly, *FLT3* mutations were classified as *FLT3*-internal tandem duplications with VAF>11(*FLT3*-ITD High VAF) and the rest. We used the following approaches for machine learning:

   1. We used RapidMiner (Boston, MA, USA) software for machine learning. A total of four classification algorithms were used to predict outcome (in this case predict overall survival) including naïve bayes, generalized linear model, deep learning and random forest methods. We used a total of 11 variables to predict outcome

(mutations in *IDH1*, *IDH2, DNMT3A* (other than R882), *DNMT3A* R882 mutations, non-ITD *FLT3* mutations, *WT1* and *TET2* as well as high *FLT3*-ITD VAF, corrected *NPM1* VAF and *NPM1* mutation type. Based on the results of these data, the global relevance of each variable was generated in terms of "weight".

2. To additionally confirm these findings, we modelled a logistic regression-based classifier to determine the relationship between the features and the probable outcome using pandas, numpy, matplotlib, sklearn as dependencies (Anaconda 3 Spyder Python 3.7). Predictor and output variables from this dataset were further divided into training and validation data sets (50% each). Models were then created for logistic regression classifier and used to generate a ROC (Receiver Operating Characteristic) curve and AUC score for both training and validation dataset. For feature selection we used SelectKBest method from sklearn feature selection module and scored features using Chi square test. The latter yielded the best features selected by calculating AUC scores for different number of features.

The performance metrics of each model with respect to accuracy, AUC & precision can be seen in Supplementary Table 3 below. In addition, we can also see the weights allotted to top 5 features for each of the ML methods used by RapidMiner.

	**Performance Metrics**			**Prediction for favourable outcome**				
	**Accuracy (%)**	**AUC (Class Alive)**	**Precision (%)**	**High Corrected *NPM1* VAF**	**Low *FLT3*-ITD VAF**	***DNMT3A* R882 WildType**	***IDH2* Mutated**	***NPM1* Mutation TypeA**
**Native Bayes**	60	0.60	69.33	0.40	0.25	0.22	0.20	0.07
**Generalized Linear Model**	63	0.61	69.33	0.34	0.32	0.12	0.17	0.09
**Deep Learning**	61	0.60	72.67	0.31	0.31	0.15	0.16	0.1
**Random Forest**	60	0.59	69.33	0.4	0.3	0.18	0.12	0.05

Supplementary Table 3: Performance metrics of different classification algorithms used for machine learning.

We developed an independent ML model using logistic regression. The performance of this model was evaluated by a confusion matrix and ROC curve. An initial pass for the training & validation dataset

that employed all variables revealed good AUC for training (0.80) but poor AUC (0.44) and accuracy (0.6) for validation datasets. To improve these metrics, we further performed feature selection and selected top predictive features with the highest AUC as seen in the supplementary table 4 below.

Number of features	AUC score
3	0.6680
4	0.7342
5	***0.7844***
6	0.7831
7	0.7837
8	0.7789

Supplementary Table 4: Feature selection and AUC Score.

Based on these data we selected top 5 features with an AUC of 0.78 (Supplementary Table 3). These five features most predictive of a patient likely to be alive were Type A *NPM1* Mutation, High Corrected *NPM1* VAF, Low *FLT3*-ITD VAF, Presence of *IDH2* Mutation, Absence of *DNMT3A* R882. These were used in training and validation datasets. The ROC curves can be seen in Figure 1 below (Accuracy 80%, Sensitivity 94.7%, Specificity 47.1%, Precision 80%)

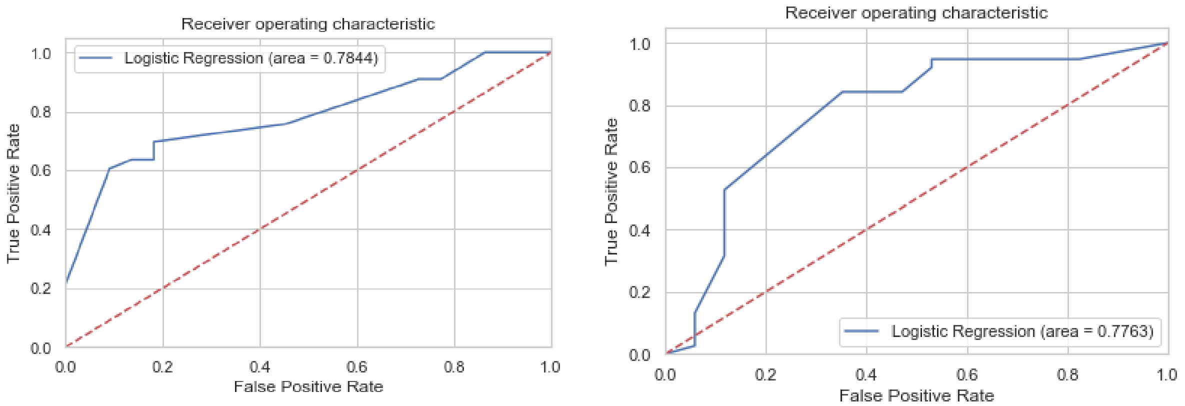

Supplementary Figure 1: The plots above show ROC of training (left) and validation (right) data sets using top 5 features.

C. Measurable Residual Disease Assessment using Multiparametric FCM (FCM-MRD):

Our FCM-MRD approach has been published previously. (2, 3) Patients who were accrued in the study from July 2012 till February 2015 were processed using an eight colour FCM assay and subsequently (till December 2018) using a 10 colour FCM-MRD assay. The presence of FCM-MRD was predictive of an inferior OS (p=0.007) and RFS (p=0.01) as seen in supplementary figure 2 and supplementary table 5.

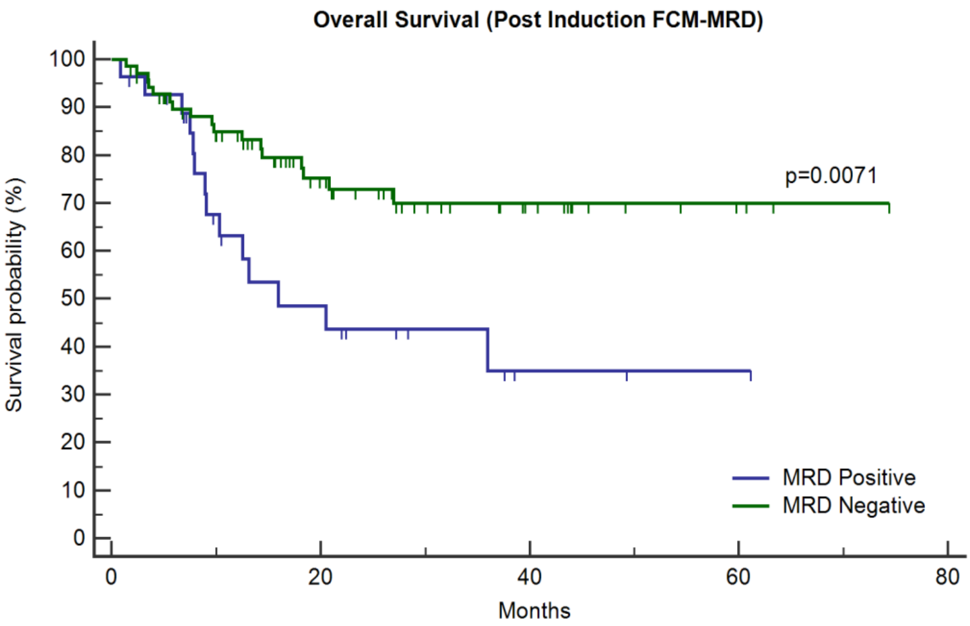

Number at risk

Group	0	20	40	60	80
Group: MRD Positive	28	10	2	1	0
Group: MRD Negative	71	33	12	3	0

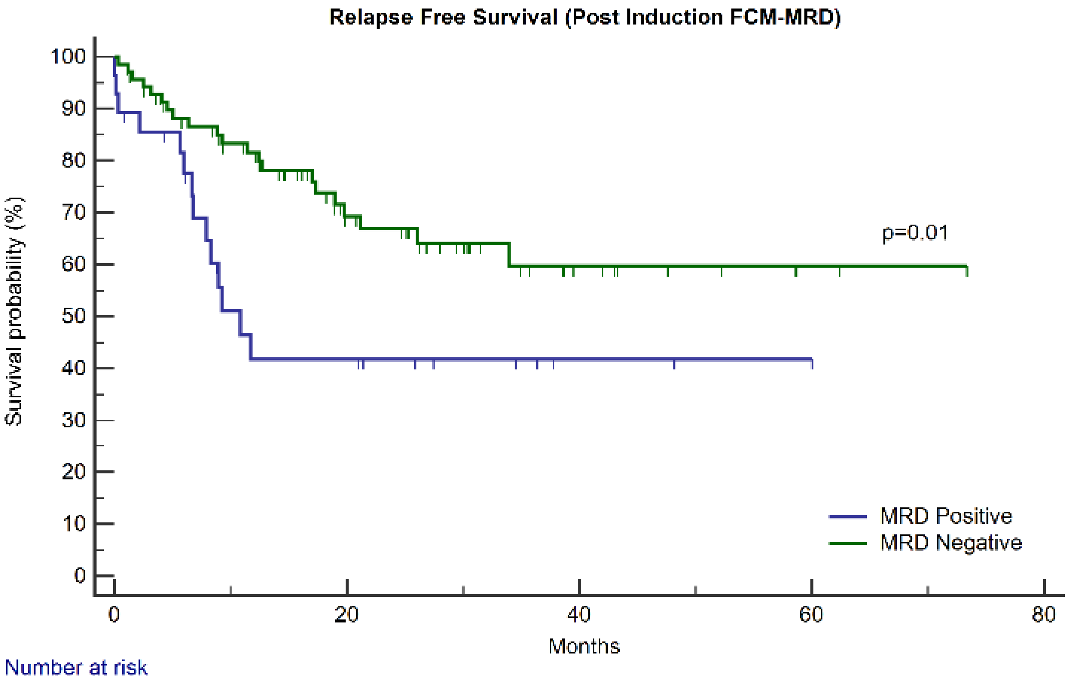

Number at risk

Group	0	20	40	60	80
Group: MRD Positive	27	9	2	1	0
Group: MRD Negative	71	29	9	2	0

Supplementary Figure 2: These Kaplan Meyer plots above show that the presence of FCM-MRD is highly predictive of inferior OS (top) and RFS (below).

D. Correlation of MRD with Genetic Risk (GR):

Patients classified as per GR defined classes were interrogated to determine differences in FCM-MRD using Chi squared test. A strong correlation was observed between ML derived genetic risk and post induction FCM-MRD as seen in supplementary figure 3.

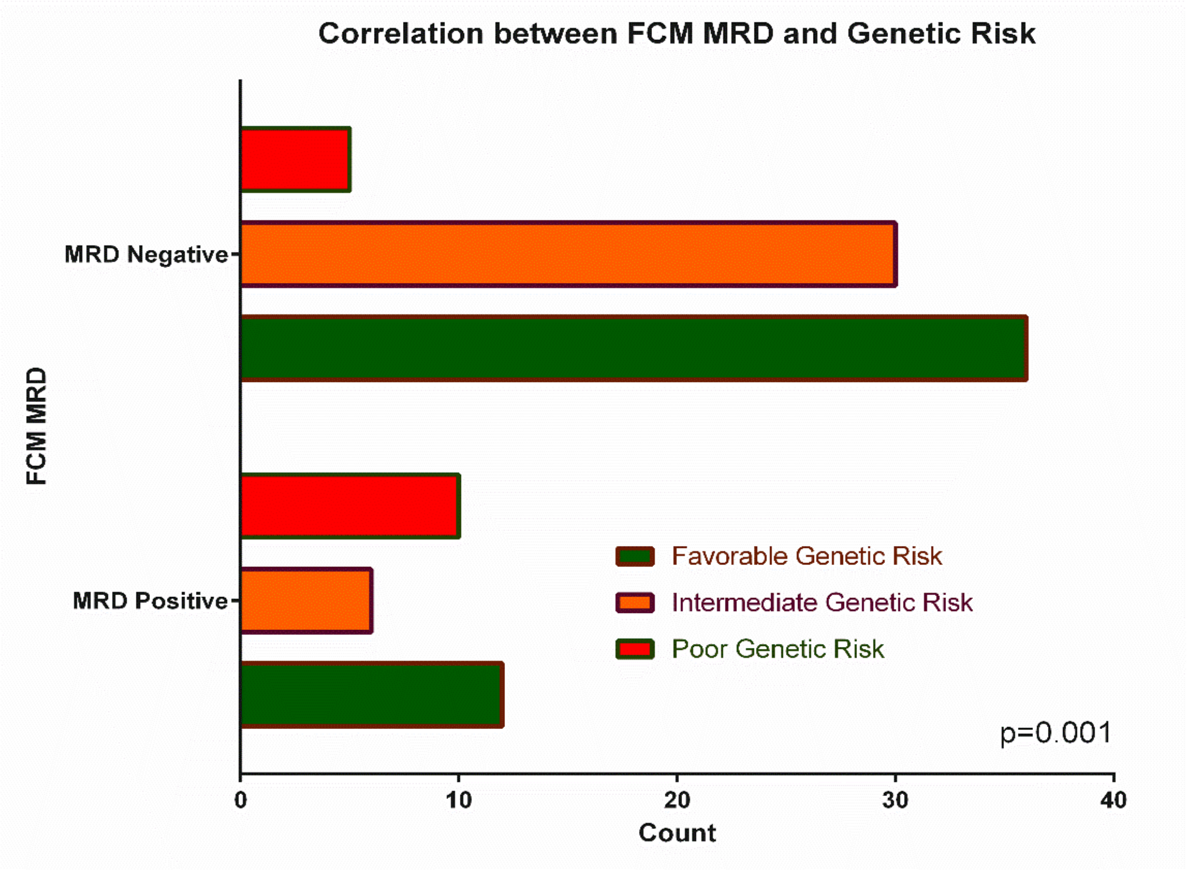

Supplementary Figure 3: A strong correlation was observed with Genetic Risk and FCM-MRD.

E. Correlation of Genetic Risk with MRD and outcome:

We analyzed results of GR as well as of FCM-MRD assays for their impact on OS and RFS using Kaplan-Meier technique and compared using log-rank test. Cox proportional-hazards regression was used to calculate hazard ratios of GR, FCM-MRD & *NPM1* NGS-MRD with univariate and multivariate analysis. MedCalc Statistical Software version 14.8.1 (MedCalc Software, Ostend, Belgium) and SPSS (IBM Corp. Released 2015. IBM SPSS Statistics for Windows, Version 23.0. Armonk, NY: IBM Corp.) were used for statistical analysis. Patients who were classified as poor

genetic risk had an inferior OS and RFS as compared to patients in favorable and intermediate risk classes as seen in supplementary table 5 below

	**Overall Survival (OS)**		**Relapse Free Survival (RFS)**	
**Post Induction FCM MRD**				
MRD Negative	Mean OS: 46.4 months; 95% CI (40.4-52.3 months),<br>Median OS: not reached		Mean RFS: 41.6 months; 95% CI (35.2-48.0 months),<br>Median RFS: not reached	
MRD Positive	Mean OS: 30.9 months; 95% CI (20.2-41.7 months),<br>Median OS: 15.9 months; 95% CI (9.1 to 35.9 months)	***p= 0.007***	Mean RFS: 27.8 months; 95% CI (18.5-41.1 months),<br>Median RFS: 10.8 months; 95% CI (7.9-11.7 months),	***p= 0.01***
**ML Derived Genetic Risk**				
Favorable GR	Mean OS: 62.3 months; 95% CI (54.7-69.9 months),<br>Median OS: not reached		Mean RFS: 57.9 months; 95% CI (49.4-66.4 months),<br>Median RFS: not reached	
Intermediate GR	Mean OS: 38.1 months; 95% CI (28.6-48.5 months)<br>Median OS: 27.0 months; 95% CI (12.5-35.9 months)	***p<0.0001***	Mean RFS: 36.5 months; 95% CI (25.7-47.2 months),<br>Median RFS: 26.0 months; 95% CI (11.7-33.9 months),	***p= 0.0003***
Poor GR	Mean OS: 12.7 months; 95% CI (6.7-18.7 months)<br>Median OS: 8.9 months; 95% CI (6.7-14.4 months)		Mean OS: 13 months; 95% CI (6.7-19.2 months)<br>Median RFS: 8.3 months; 95% CI (6.7 – 19.7 months)	

Supplementary Table 5: Difference in Overall Survival and Relapse Free Survival between FCM-MRD positive and FCM-MRD negative groups and ML derived genetic risk. OS: Overall Survival, RFS: Relapse Free Survival, CI: confidence interval, GR: Genetic Risk.

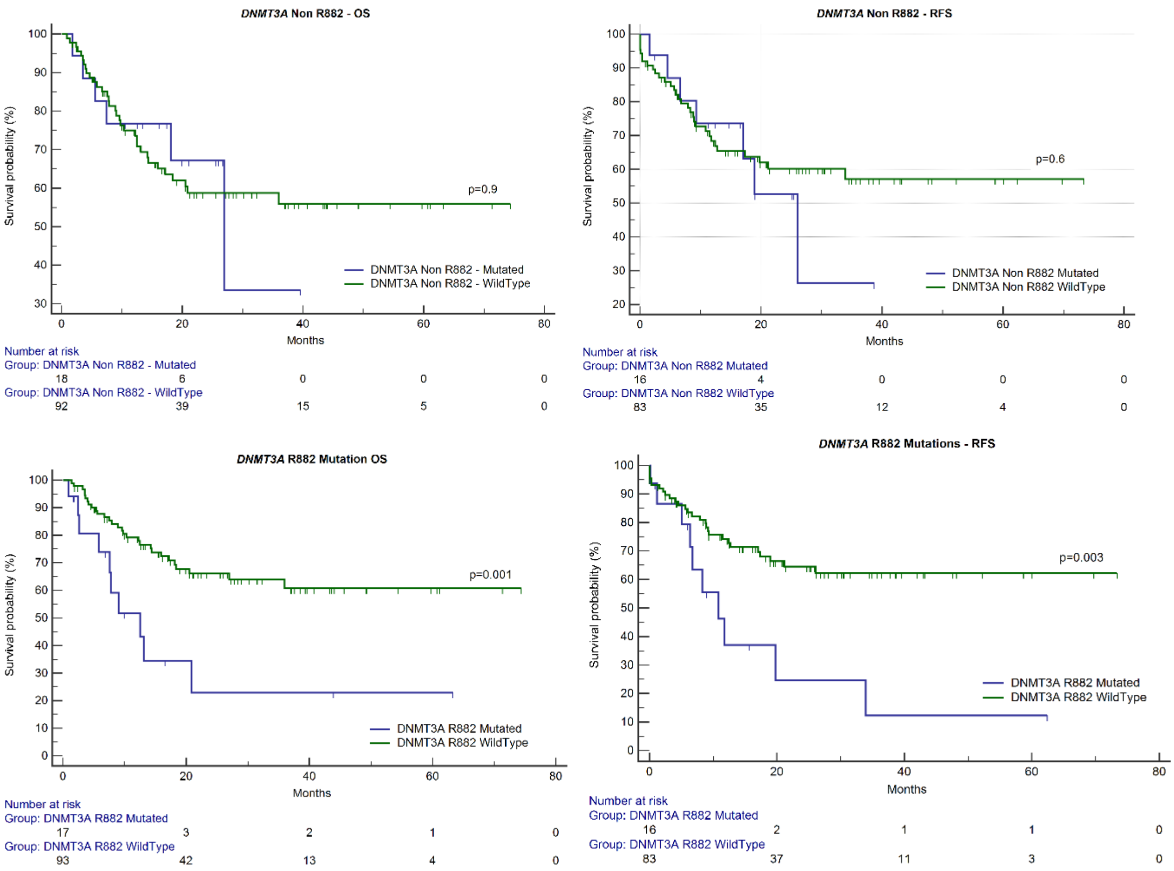

Supplementary Figure 4: Comparison of outcome (OS and RFS) between *DNMT3A* non R882 (top) and *DNMT3A* R882 mutations

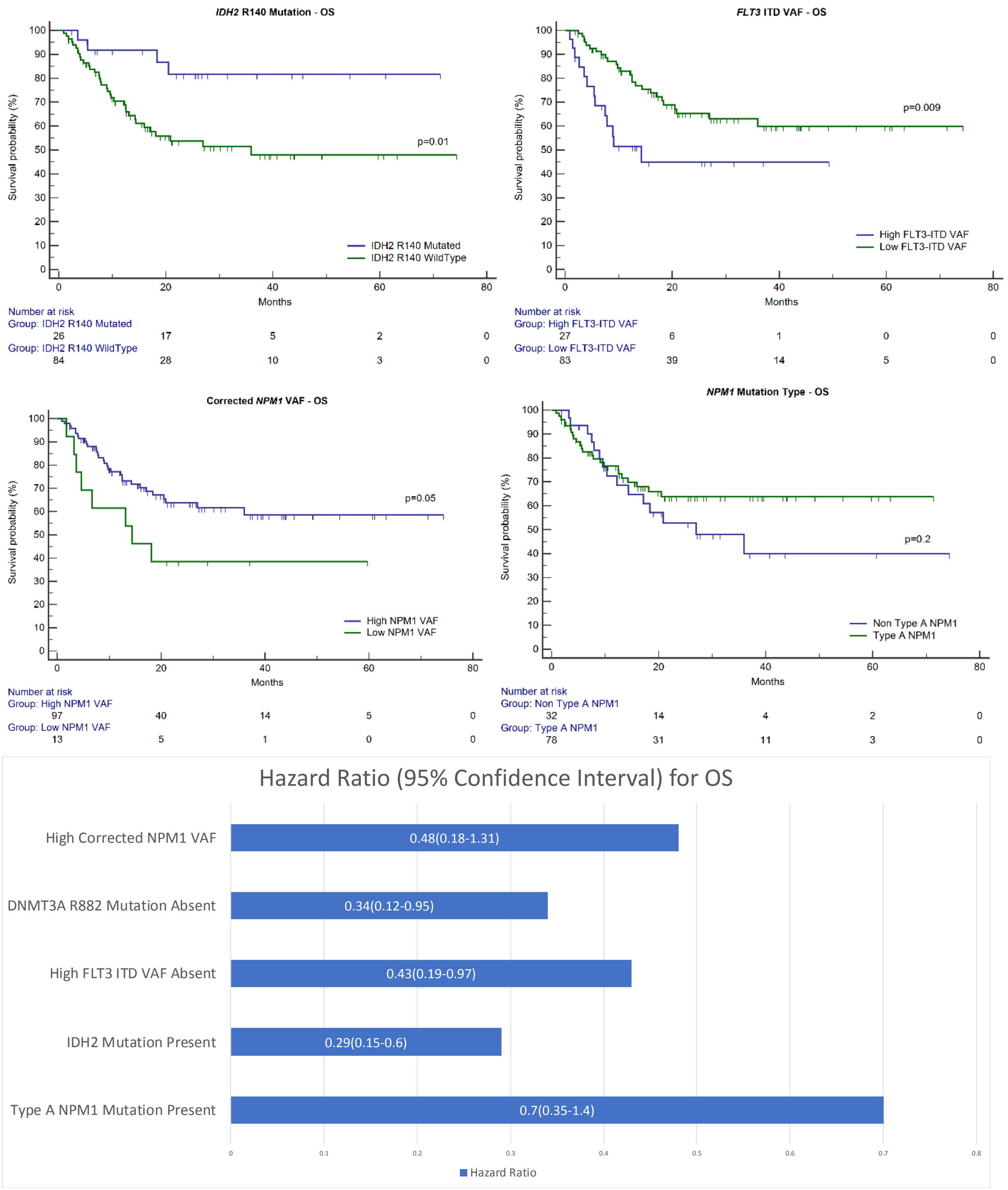

Supplementary Figure 5: Influence of *IDH2* R140 mutation, high *FLT3*-ITD VAF, corrected *NPM1* VAF and *NPM1* mutation type on outcome (OS). The bar chart at the bottom highlights hazard ratio with 95% Confidence Interval Limits

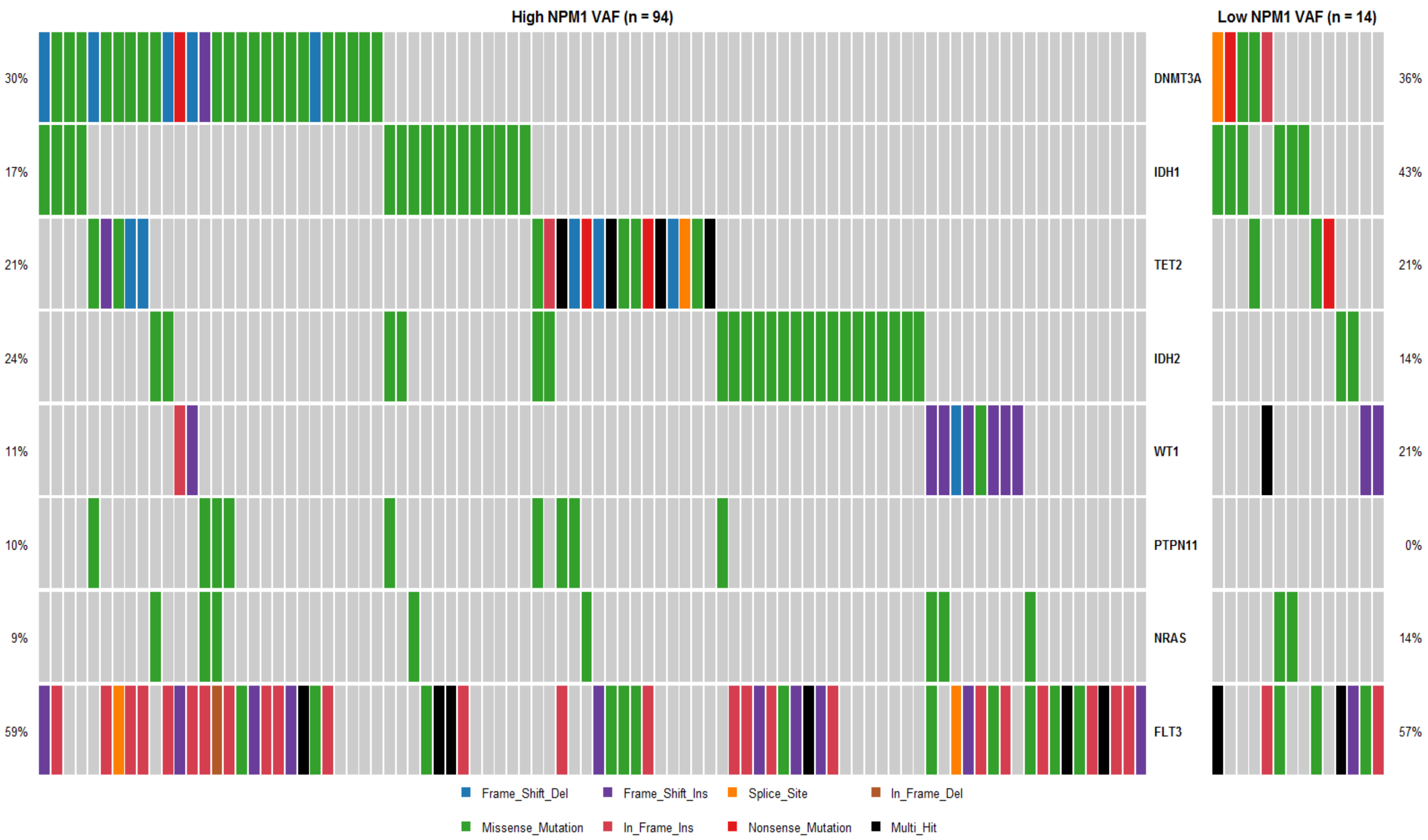

Supplementary Figure 6: Comparison of common mutations in between *NPM1* mutated AMLs divided by corrected VAF values (Two patients had isolated *NPM1* mutations and were excluded)

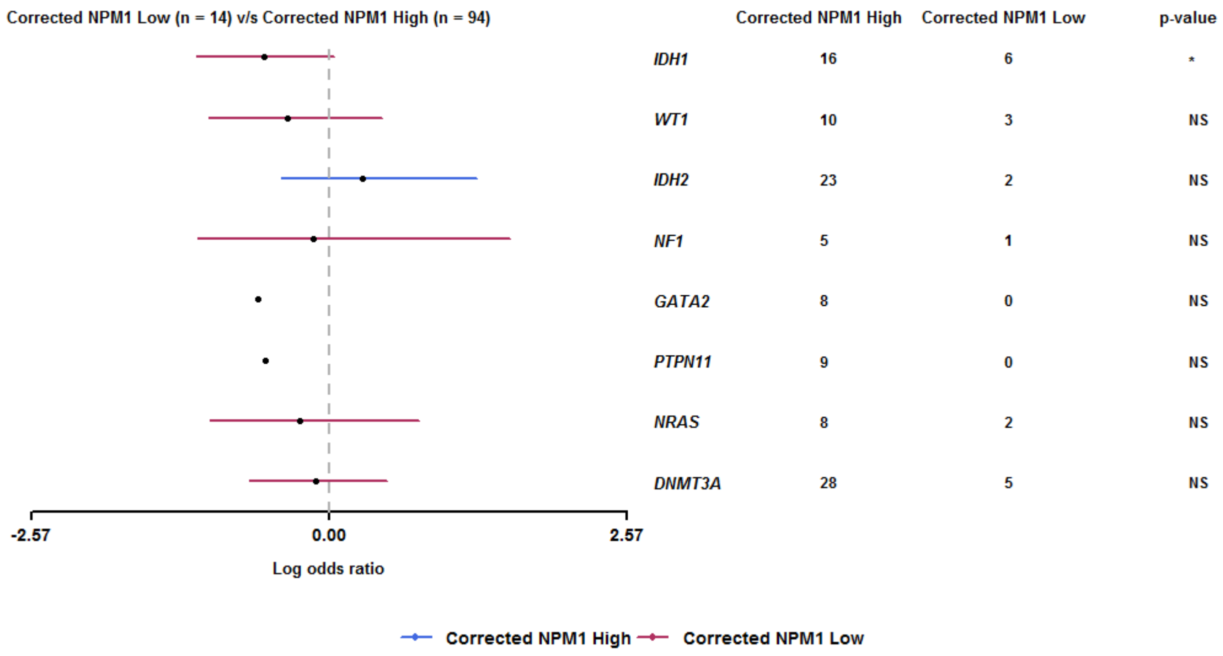

Supplementary Figure 7: Comparison of mutations in between *NPM1* mutated AMLs classified by corrected VAF values (*=0.03, NS=Not Significant)

Additional References:

1. Korf BR. Overview of clinical cytogenetics. Curr Protoc Hum Genet. 2001;Chapter 8:Unit 8.1.
2. Patkar N et al. Clinical impact of measurable residual disease monitoring by ultradeep next generation sequencing in NPM1 mutated acute myeloid leukemia. Oncotarget. 2018;9(93):36613-24.
3. Patkar N et al. Utility of Immunophenotypic Measurable Residual Disease in Adult Acute Myeloid Leukemia—Real-World Context. Frontiers in Oncology. 2019;9(450).
4. Li MM et al. Standards and Guidelines for the Interpretation and Reporting of Sequence Variants in Cancer: A Joint Consensus Recommendation of the Association for Molecular Pathology, American Society of Clinical Oncology, and College of American Pathologists. J Mol Diagn. 2017;19(1):4-23.
