# Supplementary material for: A novel machine-learning-derived genetic score correlates with measurable residual disease and is highly predictive of outcome in acute myeloid leukemia with mutated NPM1
Source: Blood Cancer J. 2019 Oct 1;9(10):79. doi: 10.1038/s41408-019-0244-2 (PMC6773777; doi:10.1038/s41408-019-0244-2)
Supplement: Supplementary file 2 — Supplementary Cytogenetics Data [file 41408_2019_244_MOESM2_ESM.pdf]

UPN	FISH	Conventional Karyotyping
NPM1	tri-tetrasomy 8	52,XX, +4, +8X2, +13,+18,+22[14] / 47-51,XX, +4[2], +8X2[3],+18[4], +22[2][cp4]
NPM2	Negative	NA
NPM3	Negative	46,XX[20]
NPM4	trisomy 8	47,XX,+8[19] / 46,XX[1]
NPM5	Negative	46,XX,del(13)(q14q21)[3] / 45,X,-X [3] / 46,XX[13]
NPM6	Negative	NA
NPM7	Negative	NA
NPM8	Negative	NA
NPM9	Negative	46-47,XY,+4[2],+9[2],+21[2] [cp5] / 46,XY [15]
NPM10	Negative	46,XX[20]
NPM11	Negative	NA
NPM12	Negative	NA
NPM13	Negative	NA
NPM14	Negative	47,XY, +21[10] / 46,XY[3]
NPM15	Negative	46,XY[15]
NPM16	Negative	NA
NPM17	Negative	NA
NPM18	Negative	NA
NPM19	Negative	45,X,-Y[10] / 45,X,-Y,t(?;9)(?;q34)[3] / 44-45,XY,-Y[3],t(9)(q34)[1] [cp3]
NPM20	Negative	NA
NPM21	Negative	NA
NPM22	Negative	NA
NPM23	Negative	NA
NPM24	Negative	NA
NPM25	Negative	NA
NPM26	Extra RUNX1T1 allele/trisomy 8	NA
NPM27	Negative	NA
NPM28	Negative	46,XY [15]
NPM29	Negative	NA
NPM30	Negative	NA
NPM31	Negative	NA
NPM32	Negative	NA
NPM33	Negative	NA
NPM34	Negative	46,XX [15]
NPM35	Negative	NA
NPM36	Negative	46,XX [12]
NPM37	Negative	NA
NPM38	Negative	46,XY [14]
NPM39	Negative	NA
NPM40	Negative	NA
NPM41	Negative	NA
NPM42	Negative	NA
NPM43	Negative	46,XX [17]
NPM44	Negative	NA
NPM45	Negative	46,XX [18]
NPM46	Negative	NA
NPM47	Negative	NA
NPM48	Negative	NA
NPM49	Negative	NA
NPM50	Negative	NA
NPM51	Negative	NA
NPM52	Negative	NA
NPM53	Negative	46,XX[20]
NPM54	Negative	NA
NPM55	Negative	NA
NPM56	Negative	NA
NPM57	Negative	46,XY[20]

NPM58	Negative	46,XY[20]
NPM59	Negative	NA
NPM60	Negative	NA
NPM61	Negative	46,XY,add(11)(p15)[7] / 46,XY[8]
NPM62	Negative	46,XY[20]
NPM63	Negative	46,XY, +9[2][cp2] / 46,XY[17]
NPM64	Negative	NA
NPM65	Negative	NA
NPM66	Negative	NA
NPM67	Negative	46,XX[20]
NPM68	Negative	46,XX[25]
NPM69	Negative	NA
NPM70	Negative	46,XX [20]
NPM71	Negative	46,XY[9]
NPM72	Negative	NA
NPM73	Negative	NA
NPM74	Negative	NA
NPM75	Negative	NA
NPM76	Negative	46,XY[20]
NPM77	NA	NA
NPM78	Negative	NA
NPM79	Negative	NA
NPM80	Negative	NA
NPM81	Negative	NA
NPM82	Negative	NA
NPM83	Negative	46,XX [18]
NPM84	Negative	46,XY [17]
NPM85	Negative	46,XX, ?inv(10) (p13;q22) [13] / 46,XX [7]
NPM86	Negative	46,XX [13]
NPM87	Negative	46,XY [16]
NPM88	Negative	NA
NPM89	Negative	46,XX[13]
NPM90	Negative	NA
NPM91	Negative	NA
NPM92	Negative	NA
NPM93	Negative	NA
NPM94	Negative	NA
NPM95	NA	45-47, XX/X, random abnormalities[cp4] / 46,XX[16]
NPM96	Negative	46-47,XX, i(Xq)[2],del(9q11)[2],del(11q23)[2],+21[3][cp7]
NPM97	Negative	NA
NPM98	Negative	NA
NPM99	Negative	46,XY, del(11)(q13)[2] / 43-46,XY,del(5q)[2], -8[3], +8[2], t(13;?)(p11;?)[2],+21[2][cp5] / 46,XY[6
NPM100	Negative	NA
NPM101	Negative	46, XX, random abnormalities [1] / 46, XX [7]
NPM102	trisomy 8, monosomy 11	44-46, XY, del (5) (q? 15/q22)[1] random aberrations[4] / 46, XY[11]
NPM103	monosomy 21	44-46,XX,/X, -X[3], -21[3],+mar1[2][cp5] / 46, XX[10]
NPM104	Negative	46,XX, del(12)(p13)[2] / 46-47,XX, -8[3], +11[2], del(11q23)[3], del(12p13)[1], +18[3], del(22)(q12)[2][cp8] / 46,XX[3]
NPM105	Negative	44-45,XY, random abnormalities[2] / 46,XY[13]
NPM106	Negative	NA
NPM107	Negative	46,XY [24]
NPM108	Negative	NA
NPM109	del(5q)	46,XX,i(3q)[3] / 46,XX, del(5)(q33)[3] / 46,XX, i(3q), del(5)(q33)[2]/ 45-46,XX, i(3q)[3], del(5q)[1],-18[3][cp4] / 46,XX[8]
NPM110	inv(16), del(7q)	NA
